# Opportunistic osteoporosis screening using multiphase abdominal CT: reliable proximal femur BMD assessment with AI-assisted segmentation

**DOI:** 10.3389/fmed.2026.1886437

**Published:** 2026-08-12

**Authors:** Fenghuan Lin, Juan Xu, Yanxia Chen, Jun Chen, Rulin Xu, Mengqiang Xiao, Xiarong Gong

**Affiliations:** 1 Department of Radiology, Zhuhai Hospital, Guangdong Provincial Hospital of Chinese Medicine, Guangdong Province, Zhuhai, China; 2 Medical Imaging Center, Qujing Central Hospital of Yunnan Province, Yunnan Province, Qujing, China; 3 Canon Medical Systems China Co Ltd, Guangdong Province, Guangzhou, China; 4 Department of MR, The First People's Hospital of Yunnan Province, The Affiliated Hospital of Kunming University of Science and Technology, Kunming, Yunnan, China

**Keywords:** artificial intelligence segmentation, contrast-enhanced CT, opportunistic screening, osteoporosis, proximal femur, quantitative CT

## Abstract

**Objectives:**

To evaluate the agreement and classification concordance of artificial intelligence (AI)-assisted proximal femur bone mineral density (BMD) assessment using multiphase contrast-enhanced abdominal computed tomography (CT), with unenhanced CT-derived quantitative computed tomography (QCT) serving as the reference standard.

**Methods:**

This retrospective study included 429 patients (mean age, 60.48 ± 12.30 years) who underwent a standardized five-phase abdominal CT protocol, comprising unenhanced, arterial, portal venous, late venous, and excretory phases. A commercially available AI-based QCT workstation was used for semi-automatic proximal femur segmentation and volumetric BMD (vBMD) and T-score calculation. Repeated-measures ANOVA and Bland-Altman analysis were performed to assess inter-phase differences and agreement. Classification concordance was evaluated using the unenhanced CT-derived QCT results as the reference standard.

**Results:**

The study cohort comprised 378 osteoporotic patients and 51 non-osteoporotic patients, categorized according to unenhanced CT-derived T-score reference values. Results from repeated-measures ANOVA indicated significant phase-related variations in T-scores and vBMD (both *P* < 0.001). Subsequent *post-hoc* comparisons revealed that T-scores and vBMD in the arterial and portal venous phases were consistent with the unenhanced phase, whereas modest yet statistically significant deviations were observed in the late venous and excretory phases. Bland-Altman analyses showed minimal mean bias across arterial, portal venous, and excretory phases; however, the late venous phase exhibited broader limits of agreement (LoA). Classification concordance compared to the unenhanced reference was consistently high across all phases, ranging from 98.14 to 99.53%. Additionally, intraobserver reproducibility remained excellent, with ICC values between 0.964 and 0.996.

**Conclusion:**

AI-assisted proximal femur BMD assessment on contrast-enhanced abdominal CT showed high agreement and classification concordance with unenhanced CT-derived QCT. The arterial and portal venous phases were particularly suitable for opportunistic osteoporosis screening, although external validation against DXA or independent QCT remains necessary.

## Introduction

The reduction in bone mineral density (BMD) and fracture predisposition is known as osteoporosis and poses a major public health problem in older adults. Fractures resulting from osteoporosis incur significant health care expenditure (1, 2). It is important that fractures should be diagnosed at early stages for the effective management and prevention of the morbidities associated with them.

Though dual-energy X-ray absorptiometry (DXA) is considered the gold standard for assessing BMD, the two dimensional imaging system of DXA has some limitations. They are prone to errors due to degenerative changes in spine such as osteophytes, sclerosis, and vascular calcifications (3). On the other hand, QCT provides the three dimensional measurement of trabecular bones with the interference of the cortical bone being minimized. Thus, this method is especially beneficial in patients who have lost significant amount of their cancellous bone (4, 5). The 2015 guidelines provided by International Society for Clinical densitometry state that femoral neck and total hip T-scores obtained by QCT are reliable indicators of osteoporosis when proper reference standards are used (6).

While QCT is very precise, a relatively high radiation dose limits its clinical application due to this characteristic. Opportunistic BMD testing through the existing CT exams is very valuable from the clinical point of view since this approach makes it possible to diagnose osteoporosis without an additional radiation dose and prolongation of examination time. Currently, opportunistic BMD testing is conducted mostly during the low-dose non-contrast chest CT (7, 8). At the same time, abdominal CT studies, including the contrast-enhanced protocol that is currently widely conducted without a non-contrast phase (9), are an unexplored area of osteoporosis screening. Since the lumbar spine and proximal femur are anatomically defined areas to evaluate BMD (10), adding the opportunistic BMD measurement to multiphase contrast-enhanced abdominal CT can be extremely useful.

As far as the available literature goes, there have been a few studies exploring the feasibility and accuracy of the opportunistic bone mineral density quantification using the contrast-enhanced abdominal CT, with some inconsistencies in their results (11, 12). The technological improvements have allowed obtaining direct imaging with the use of the contrast enhancement in order to obtain the diagnostic performance, which is equivalent to the one of the combined approach in certain clinical applications, namely lower-extremity vascular imaging, liver disease assessment and abdominal CT scans (13, 14). Nevertheless, doubts exist with respect to the precision of the measurements of BMD during the contrast-enhanced phases. The past researches have mainly focused on single and dual-phase contrast-enhanced images and the majority of the studies were performed for the lumbar spine. In more recent studies performed among both adults and children after liver transplants, it has been demonstrated that the attenuation values of vertebrae obtained from regular contrast-enhanced abdominal CT correspond to the DXA BMD values (15, 16).

Considering the above-mentioned background, the objective of the current research work is to systematically assess opportunistic BMD measures taken from multiphase contrast-enhanced abdominal CT scans that consist of arterial, portal venous, late venous, and excretory phases in a large group of patients. In order to enhance the measurement accuracy and minimize the variations associated with manual segmentation, artificial intelligence (AI)-based segmentation procedure was utilized for the proximal femur region. The inter-phase consistency, agreement, and reproducibility of the measurements were determined using unenhanced CT QCT as an internal reference standard.

## Materials and methods

### Study population

This retrospective study received ethical approval from the institutional ethics committee (approval number: ZE2024-243), and the requirement for informed consent was waived.

**Figure 1:**
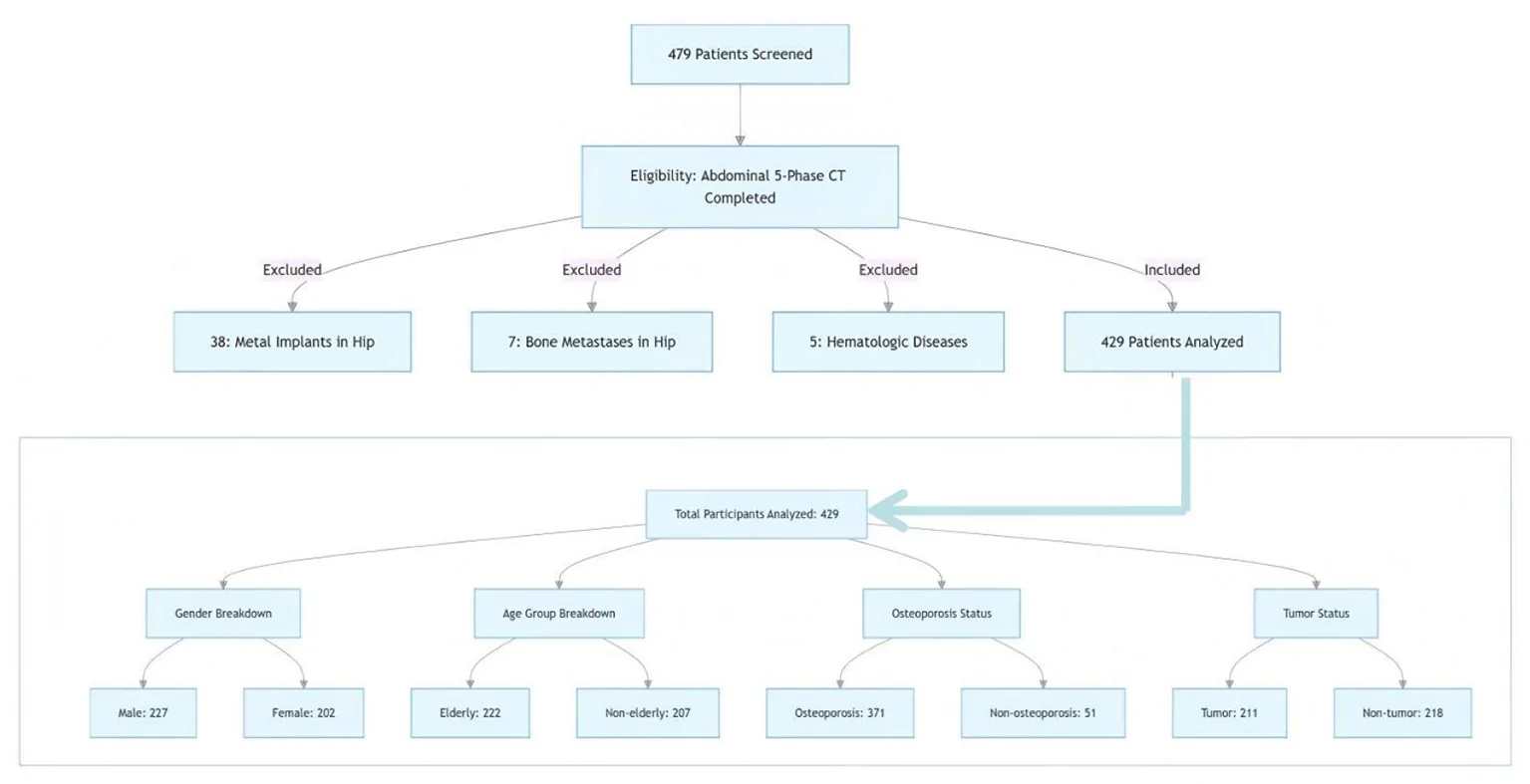
Study flow diagram.

Initially, 479 consecutive patients undergoing five-phase abdominal CT between February 2022 and December 2024 were screened. Patients were excluded based on the following criteria: presence of hip fractures; hip pathology involving foreign materials or morphological alterations (including benign or malignant bone lesions); metallic implants near the hip joint (*n* = 38); known hip-related malignancies, such as bone metastases (*n* = 7); and hematologic bone disorders (*n* = 5). Ultimately, 429 patients (227 women and 202 men; mean age, 60.48 ± 12.30 years) met inclusion criteria and were analyzed (Figure 1).

### CT acquisition and contrast-enhanced protocol

All imaging studies were conducted using a 320-row detector CT scanner (Aquilion ONE; Canon Medical Systems, Japan), following established quality assurance protocols conducted daily, weekly, and monthly to maintain consistent Hounsfield unit measurements. The standardized imaging protocol encompassed an unenhanced scan followed by four contrast-enhanced phases: arterial phase at 30 ± 2 s, portal venous phase at 63 ± 2 s, late venous phase at 4 min, and excretory phase at 30 min. The excretory-phase scan was confined to the pelvic region. Imaging parameters included a 200 mm field of view, reconstruction slice thickness of 0.5 mm with 0.5 mm intervals, a tube voltage of 120 kVp, and automated tube current modulation. A low-osmolar nonionic iodinated contrast medium (iopromide 370 mg I/mL; Ultravist, Bayer Healthcare, Leverkusen, Germany) was administered intravenously at a flow rate of 3–4 mL/s, totaling 80–90 mL and an iodine dosage of 450 mg I/kg.

### BMD assessment

Reconstructed datasets from the five CT phases were transmitted to an asynchronous QCT workstation (iCare QCT; Junlang Technology Co., Ltd., Changsha, China) specifically for assessing proximal femur BMD. This software, commercially available and approved by China's National Medical Products Administration, has demonstrated validation through phantom-based studies (17).

**Figure 2:**
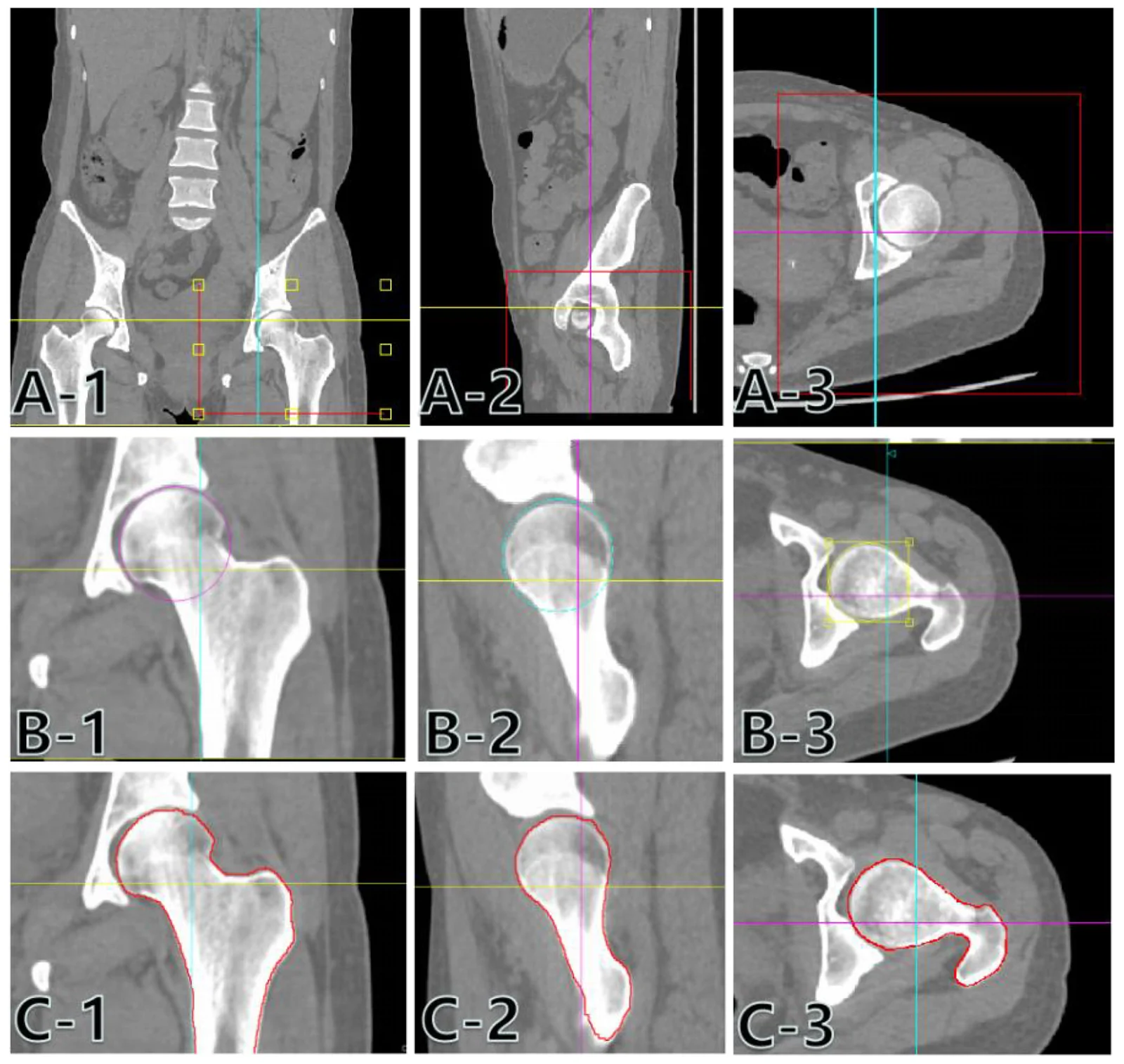
Workflow for volumetric bone mineral density assessment on hip CT scans. **(A1, A2 and A3)** show raw CT images of the hip joint. In **(B1, B2 and B3)**, the femoral Q22 head is highlighted using a circular marker; a single click on the acetabular cortical bone activates the AI-based segmentation algorithm, which automatically delineates the three-dimensional proximal femur from the superior margin of the femoral head to 1 cm below the greater and lesser trochanters. **(C1, C2 and C3)** present the automatically generated volumetric BMD and corresponding T-scores for the defined region of interest.

The workstation employs a semi-automated AI-driven algorithm for femoral segmentation. After initial manual landmark placement, the software delineates the proximal femur in three dimensions, covering the femoral head, neck, and extending approximately 1 cm below the greater and lesser trochanters (Figure 2). An experienced radiologist subsequently reviewed and verified the segmentation visually. The vBMD and corresponding T-scores were automatically calculated using an integrated, sex-specific young-adult normative database. Osteoporosis classification adhered to standard T-score thresholds: normal (T-score ≥ −1.0), osteopenia (−2.5 < T-score < −1.0), and osteoporosis (T-score ≤ −2.5) (6). For concordance analyses, individuals categorized as normal and osteopenic were grouped as non-osteoporotic.

All measurements were repeated twice by the same radiologist at an interval of 15 days, with mean values utilized for primary analysis. The repeated measurements assessed intraobserver reproducibility.

### Statistical analysis

Statistical analyses were executed using IBM SPSS Statistics (version 27.0; IBM Corp., Armonk, NY, USA). Continuous variables underwent normality testing via the Shapiro–Wilk test and are expressed as mean ± standard deviation (SD). Categorical data are presented as counts and percentages.

Repeated-measures ANOVA compared T-scores and vBMD across all five CT phases within patients. Sphericity was evaluated by Mauchly's test, with Greenhouse-Geisser corrections applied when violations occurred. Bonferroni-adjusted pairwise *post-hoc* comparisons assessed differences between each contrast-enhanced phase and the unenhanced reference.

Agreement between each contrast-enhanced phase and the unenhanced reference was evaluated through Bland-Altman analyses, calculating mean biases and 95% limits of agreement (LoA), defined as mean bias ± 1.96 × SD of paired differences.

Intraobserver reproducibility was assessed via intraclass correlation coefficient (ICC), calculated using a two-way mixed-effects, absolute-agreement model. Classification concordance represented the proportion of consistent osteoporosis categorizations between each contrast-enhanced phase and the unenhanced reference. Subgroup analyses were stratified by age ( ≤ 60 vs. >60 years), sex, osteoporosis status, and malignancy history. Statistical significance was set at a two-sided *P* value < 0.05.

### Results

Among 429 included patients, 51 were non-osteoporotic (2 normal, 49 osteopenic), and 378 were osteoporotic based on unenhanced CT-derived T-score criteria. The patient cohort included 211 individuals with malignancy histories, notably colorectal cancer (*n* = 87), gynecologic tumors (*n* = 26), hepatobiliary tumors (*n* = 22), pancreatic tumors (*n* = 18), gastric tumors (*n* = 19), and other abdominal malignancies (*n* = 39), while 218 patients had no oncologic history.

High intraobserver reproducibility was observed across all phase-specific T-score and vBMD measurements (ICC values: 0.964–0.996).

Significant differences were noted across phases for both T-scores (*F* = 64.57, *P* < 0.001) and vBMD (*F* = 40.83, *P* < 0.001) using repeated-measures ANOVA. Greenhouse-Geisser corrections were applied due to sphericity violations. Bonferroni-corrected *post-hoc* tests indicated no significant differences between arterial or portal venous phases and the unenhanced phase (*P* > 0.05), whereas minor yet statistically meaningful differences were observed in late venous and excretory phases compared to the unenhanced reference (*P* < 0.05).

**Figure 3:**
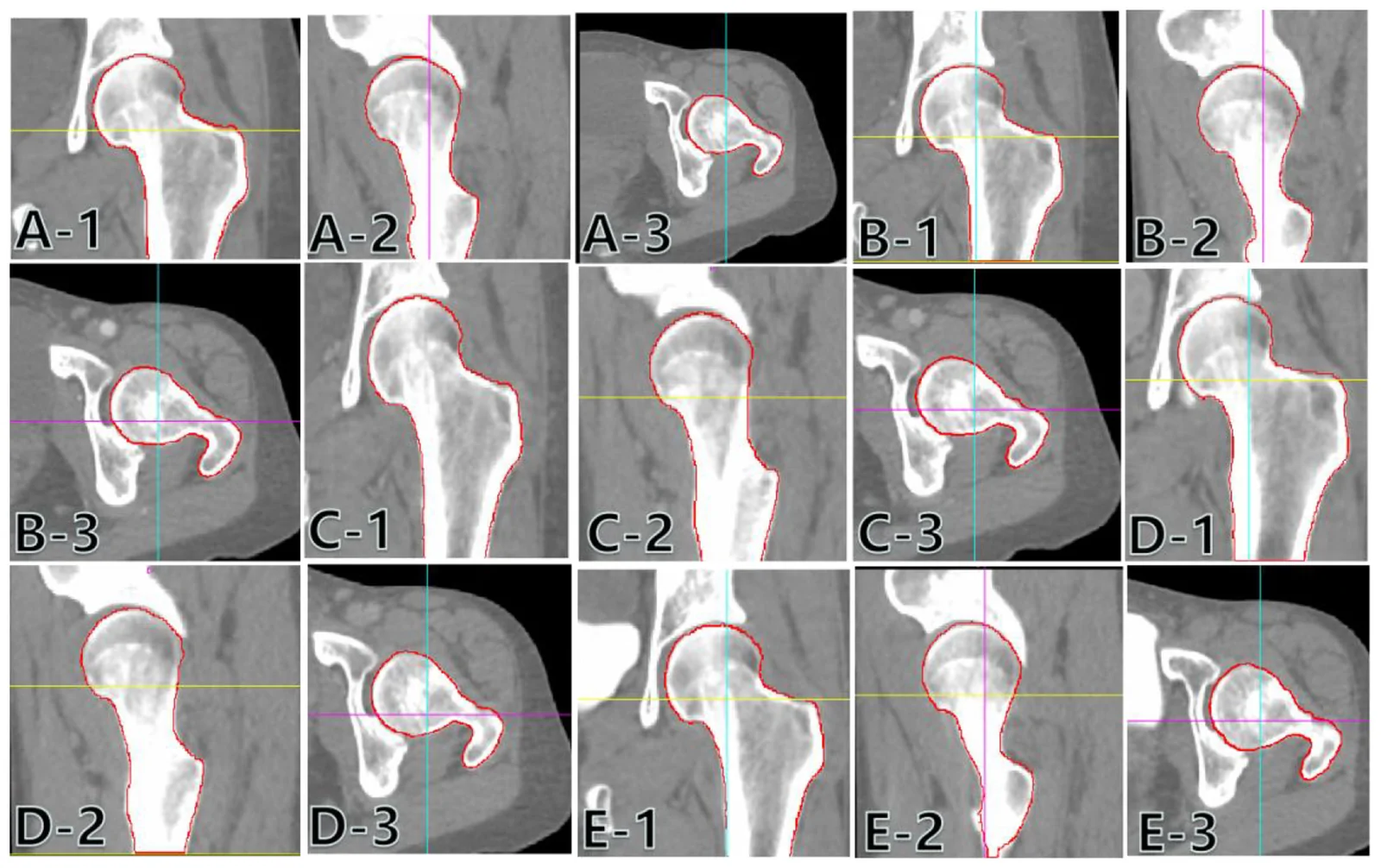
Representative BMD and *T*-value measurements in a 46-year-old female patient. **(A-1, A-2, A-3)** Plain (unenhanced) CT: *T*-value = −1.115; BMD = 359.27 mg/cm^3^. **(B-1, B-2, B-3)** Arterial phase: *T*-value = −1.066; BMD = 361.68 mg/cm^3^. **(C-1, C-2, C-3)** Venous phase: *T*-value = −1.040; BMD = 360.00 mg/cm^3^. **(D-1, D-2, D-3)** Delayed (late venous) phase: *T*-value = −1.016; BMD = 364.18 mg/cm^3^. **(E-1, E-2, E-3)** Excretory phase: *T*-value = −1.112; BMD = 359.39 mg/cm^3^.

Across the five phases, mean T-scores were −3.54 ± 0.98 (unenhanced), −3.60 ± 0.99 (arterial), −3.57 ± 0.98 (portal venous), −3.55 ± 1.02 (late venous), and −3.65 ± 1.01 (excretory). Corresponding mean vBMD values were 235.43 ± 49.26, 233.76 ± 48.04, 235.93 ± 47.67, 232.97 ± 42.67, and 231.54 ± 48.25 mg/cm^3^, respectively (Figure 3; Table 1).

**Table 1:** T-score and vBMD measurements across five CT phases in the overall cohort (*n* = 429).

Phase	Mean *T*-value ±SD (*n* = 429)	Mean BMD ±SD (mg/cm^3^) (*n* = 429)
Unenhanced	−3.540 ± 0.98	235.434 ± 9.26
Arterial	−3.600 ± 0.99	233.764 ± 8.04
Portal venous	−3.570 ± 0.98	235.934 ± 7.67
Late venous	−3.551 ± 0.02	232.974 ± 2.67
Excretory	−3.651 ± 0.01	231.544 ± 8.25

Values are expressed as mean ± SD. Repeated-measures ANOVA demonstrated significant overall phase effects for both T-score (*F* = 64.57, *P* < 0.001) and vBMD (*F* = 40.83, *P* < 0.001). *Post-hoc* Bonferroni-adjusted comparisons indicated that the arterial and portal venous phases did not differ significantly from the unenhanced phase (*P* > 0.05), whereas the late venous and excretory phases showed small but statistically significant differences (*P* < 0.05).

Bland-Altman analysis indicated small mean biases for the arterial, portal venous, and excretory phases relative to the unenhanced phase. In contrast, the late venous phase demonstrated wider 95% LoA, particularly for vBMD (Table 2).

**Table 2:** Bland-Altman agreement between contrast-enhanced phases and the unenhanced CT-derived QCT reference phase.

Comparison group	SD	95% limits of agreement	Proportion of data points within 95%	Consistency grade
T (A/U)	0.06	−0.83 ~ 0.95	98.60%	Excellent
T (V/U)	0.03	−0.86 ~ 0.91	98.37%	Excellent
T (D/U)	−0.39	−2.41 ~ 1.63	94.87%	Moderate
T (E/U)	0.11	−0.96 ~ 1.18	97.67%	Good
BMD (A/U)	1.67	−37.96 ~ 41.30	99.07%	Excellent
BMD (V/U)	−0.5	−38.49 ~ 37.48	98.83%	Excellent
BMD (D/U)	−15.82	−120.97 ~ 89.34	96.04%	Moderate
BMD (E/U)	3.88	−42.63 ~ 50.39	97.90%	Good

LoA, limits of agreement; vBMD, volumetric bone mineral density.

In the arterial phase, five of 51 non-osteoporotic patients were misclassified as osteoporotic, and three of 378 osteoporotic patients were misclassified as non-osteoporotic, resulting in a classification concordance of 98.14%. In the portal venous phase, three non-osteoporotic cases and five osteoporotic cases were misclassified, yielding the same concordance of 98.14%. In the late venous phase, two non-osteoporotic cases and 1 osteoporotic case were misclassified, corresponding to a concordance of 99.30%. In the excretory phase, two non-osteoporotic cases were misclassified, while all osteoporotic cases were correctly classified, resulting in the highest concordance of 99.53%.

**Figure 4:**
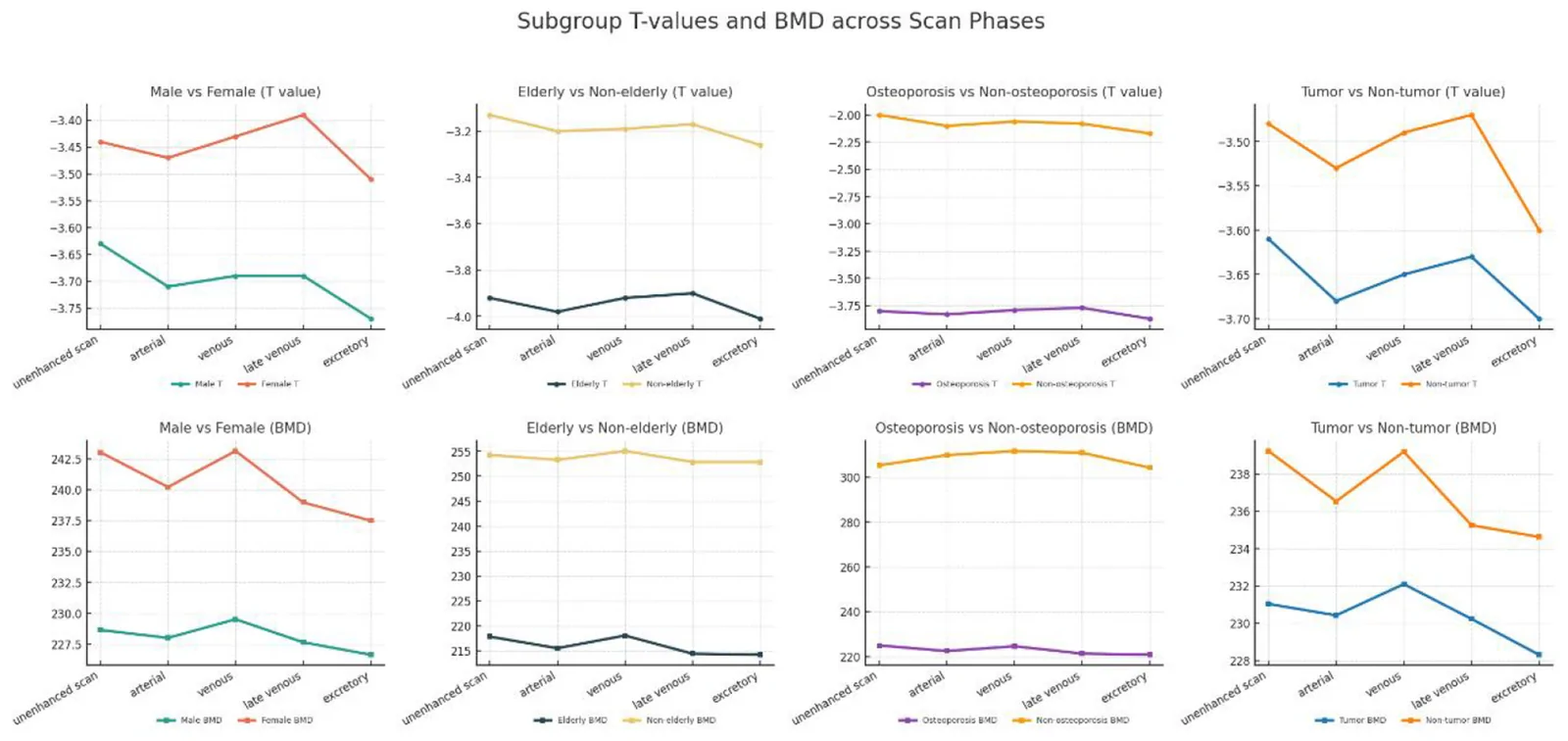
Subgroup *T*-values and BMD across scan phases. Subgroup analyses were conducted according to unenhanced CT-derived QCT classification (osteoporosis vs. non-osteoporosis), age ( ≤ 60 vs. >60 years), sex, and malignancy history. Overall, the arterial and portal venous phases demonstrated relatively stable T-score and vBMD values compared with the unenhanced reference phase, whereas the late venous and excretory phases exhibited greater phase-related variability in certain subgroups. These findings were consistent with the overall repeated-measures analysis.

Subgroup analyses by age, sex, osteoporosis status, and malignancy history confirmed consistent phase-related trends, with arterial and portal venous phases showing stable agreement with the unenhanced reference across all subgroups (Figure 4).

## Discussion

In this study, we found that multiphase contrast-enhanced abdominal CT enables opportunistic osteoporosis screening at the proximal femur when combined with AI-assisted QCT analysis. Relative to the unenhanced CT-derived QCT reference, the arterial and portal venous phases demonstrated minimal bias and strong agreement for both T-scores and vBMD. In contrast, the delayed (late venous) phase exhibited wider LoA, indicating that measurements obtained during this phase should be interpreted with increased caution. While the excretory phase had a positive correlation, the utility of the phase in the clinical setting might be limited due to the absence of routine performance in standard abdominal CT scan procedures. Overall, the study showed that the use of intravenous contrast did not significantly hinder BMD evaluation of the proximal femur bones in regular abdominal CT scans, particularly during the arterial and portal venous phases. Essentially, patients scheduled for contrast-enhanced abdominal or pelvic CT scan can be considered for osteoporosis screening without being subjected to further radiation dose, prolonged scanning, or specific QCT studies (18).

It is noteworthy that the frequency of osteoporosis among the group participants was quite high, since out of 429 subjects 378 were classified as suffering from this disease by the unenhanced CT reference of QCT. The observed ratio is expected to reflect the clinical features of the study participants, because they were elderly subjects who had multiphase abdominal CT performed, many of them having been diagnosed with abdominal malignancies. The influence on bones might be caused by cancer-related features, age, lack of nutrition, sedentary lifestyle, as well as anticancer therapy. Despite the consistency of results obtained for different subgroups by phases, the rather small number of subjects without osteoporosis may still be considered a limitation.

Earlier research on opportunistic osteoporosis using contrast-enhanced CT scans has generated conflicting results, possibly because of differences in enhancement phase, anatomical location, assessment methods, and control points (19). For instance, Boutin et al. found an increase in vertebral CT attenuation during the arterial, portal venous, and delayed phases, confirming the need for phase-specific correction measures (20). In contrast, Lee et al. found insignificant change in the Hounsfield units of the proximal femur in the portal venous phase, implying that there is no need for correction in femoral bone mass measurement, although the sample size used by Lee et al. was small (21). The present findings support the notion that the effect of contrast enhancement on opportunistic BMD evaluation is site-dependent. Compared with vertebral trabecular bone, the proximal femur appears less sensitive to contrast-induced attenuation changes, which may partly explain the relatively stable T-scores and vBMD values observed in the arterial and portal venous phases.

Phase-specific variability in the current study can be explained by the time-related distribution of the iodinated contrast agent. At the arterial and portal venous phases, contrast effects in the proximal femur marrow compartment seem to have minimal effect on QCT-based BMD values. On the other hand, LoA at the delayed phase are the most extensive, probably due to a higher degree or heterogeneity of the contrast persistence in the soft and marrow compartments. Excretory phase is characterized by a relatively low impact of the contrast, resulting in good agreement with the delayed phase. Nevertheless, since the excretory phase is not included in standard abdominal CT examinations, arterial and portal venous phases have more clinical significance.

The application of AI-based segmentation is one of the primary methodological strengths of this research work. The automatic or semi-automatic identification of the proximal femur eliminates human error, increases work efficiency, and allows for standardized assessment of the volume of the femoral head, femoral neck, and proximal femur. In addition, an analysis of the whole proximal femur can better reflect the general state of bones compared to the analysis restricted to only the femoral neck (17, 22–25). However, the effectiveness of the AI-based segmentation needs to be evaluated in relation to a particular QCT device used in this study. Further external validation across different scanner models, reconstruction settings, contrast protocols, and patient populations remains necessary.

Several limitations should be acknowledged. First, it is a single center retrospective study, and the gold standard used is based on the unenhanced phase of the same CT scan, and not on the external DXA or QCT measurements. Thus, the study mainly represents the agreement between different phases, but not external validity, and its results cannot be taken as the definitive proof of the superiority of DXA. Second, the study has an unbalanced distribution, with a relatively small group of patients without osteoporosis. It might limit the ability to perform classifications-related analysis. Third, the type of tumors was known, but there was no information on the metastasis presence, chemotherapy, endocrine or targeted treatments, steroid usage and nutritional state–these might affect the BMD. Fourth, there was no systematic evaluation of the type of the contrast agent, concentration of iodine, injection rate, patient's hemodynamic status and the timing of the scan. Fifth, all analyses were done using one CT system and one QCT software; thus, the multi-center prospective studies with various vendors and reference standards are needed.

In conclusion, multiphase contrast-enhanced abdominal CT scanning can serve as an effective foundation for incidental BMD estimation of proximal femur using AI-assisted QCT. Out of all the phases, arterial and portal venous phases have shown the highest clinically significant correlation with the values of unenhanced CT QCT parameters, making them suitable candidates for incidental osteoporosis screening through abdominal CT. Delayed phase must be interpreted with caution because of higher LoA, while although excretory phase has shown excellent agreement, it might not be practical in routine settings. Further multicenter studies with external validation of DXA are required.

## Data Availability

The original contributions presented in the study are included in the article/supplementary material, further inquiries can be directed to the corresponding author/s.
